# Diffusion along perivascular spaces provides evidence interlinking compromised glymphatic function with aging in Parkinson's disease

**DOI:** 10.1111/cns.13984

**Published:** 2022-10-02

**Authors:** Xin Cai, Zhenzhen Chen, Chentao He, Piao Zhang, Kun Nie, Yihui Qiu, Limin Wang, Lijuan Wang, Ping Jing, Yuhu Zhang

**Affiliations:** ^1^ Department of Neurology, Guangdong Neuroscience Institute, Guangdong Provincial People's Hospital Guangdong Academy of Medical Sciences Guangzhou China; ^2^ The Second School of Clinical Medicine Southern Medical University Guangzhou China; ^3^ Department of Neurology Shenzhen Samii Medical Center Shenzhen China; ^4^ Department of Neurology, The Central Hospital of Wuhan, Tongji Medical College Huazhong University of Science and Technology Wuhan China

**Keywords:** aging, diffusion tensor imaging, glymphatic system, Parkinson's disease, sleep disorders

## Abstract

**Aims:**

The aim of the study was to evaluate the glymphatic function and its related factors in patients with Parkinson's disease (PD) and patients with PD of different ages using the diffusion tensor image analysis along the perivascular space (DTI‐ALPS) method.

**Methods:**

Medical records and imaging data of 93 patients with idiopathic PD and 42 age‐ and sex‐matched healthy controls (HCs) were retrospectively reviewed and analyzed. The diffusivity along the perivascular spaces, projection fibers, and association fibers were calculated on diffusion tensor imaging (DTI) to acquire the analysis along the perivascular space (ALPS) index.

**Results:**

PD patients exhibited a reduced ALPS index compared with the HCs. Negative correlations between the ALPS index and clinical information including age, age at disease onset, Parkinson's disease sleep scale 2nd version (PDSS‐2) scores, and history of diabetes mellitus were revealed in the PD group. Besides, a negative correlation between the ALPS index and the severity of motor symptoms was identified in the subgroup aged 65 and above, rather than in the younger ones.

**Conclusions:**

The results demonstrate that reduced ALPS index, a potential noninvasive measure of compromised glymphatic activity, is involved in the pathophysiology of PD, especially in the aged ones and those with sleep disorders.

## Graphical abstract

Using the noninvasive diffusion tensor image analysis along the perivascular space (DTI‐ALPS) method, this study revealed compromised glymphatic circulation and its significant associations with age, age at disease onset and sleep disorders in Parkinson's disease and supported that glymphatic function may underpin the involvement of aging in the pathophysiology of Parkinson's disease.

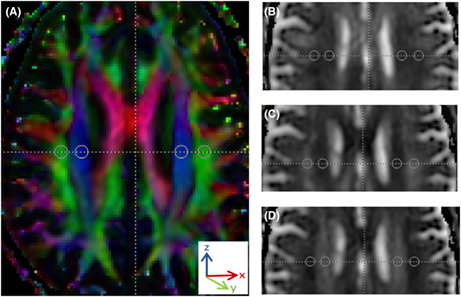

## 1 INTRODUCTION

Parkinson's disease (PD) is the second most common neurodegenerative disease.^1^ It is heterogeneous in clinical manifestations and progression, and related pathophysiology has been explored only to a limited extent.^2^ Major neuropathological changes in PD patients involve aggregation and propagation of misfolded α‐synuclein.^3^ Age is widely accepted as a major risk of PD.^4^ And it has been reported that the accumulation of α‐syn in the brain is greatly related to age.^5^ However, the underlying mechanism between aging and the pathogenesis and progression of PD is poorly understood.^6^, ^7^, ^8^

The glymphatic (glial‐lymphatic) pathway was first identified in rodent brains.^9^ In the glymphatic system, cerebrospinal fluid (CSF) exchanges with interstitial fluid (ISF) and then drains extracellular soluble proteins and metabolites along the perivascular spaces into the cervical lymphatic vessels.^10^, ^11^, ^12^ This process is mainly driven by arterial pulsation and mass flow facilitated by astroglial water channel aquaporin 4 (AQP4),^9^, ^11^ and is with robust activity during sleep.^9^, ^13^, ^14^ Commonly shared by PD patients and the aged population, sleep disorders contribute to decreased glymphatic clearance. In addition, aging has been reported to be associated with a steep fall in glymphatic flow in the brains of both rodents and humans.^15^, ^16^ Although α‐synuclein mainly deposits intracellularly, samples of CSF and extracellular fluid have documented α‐synuclein outside the cytosol.^17^, ^18^ Moreover, the transmission of α‐synuclein involves the extracellular pathway.^19^ Therefore, glymphatic dysfunction may contribute to decreased clearance of α‐synuclein and provide the construct with the involvement of aging and sleep disorders in the pathogenesis and progression of PD.^20^

**FIGURE 1:**
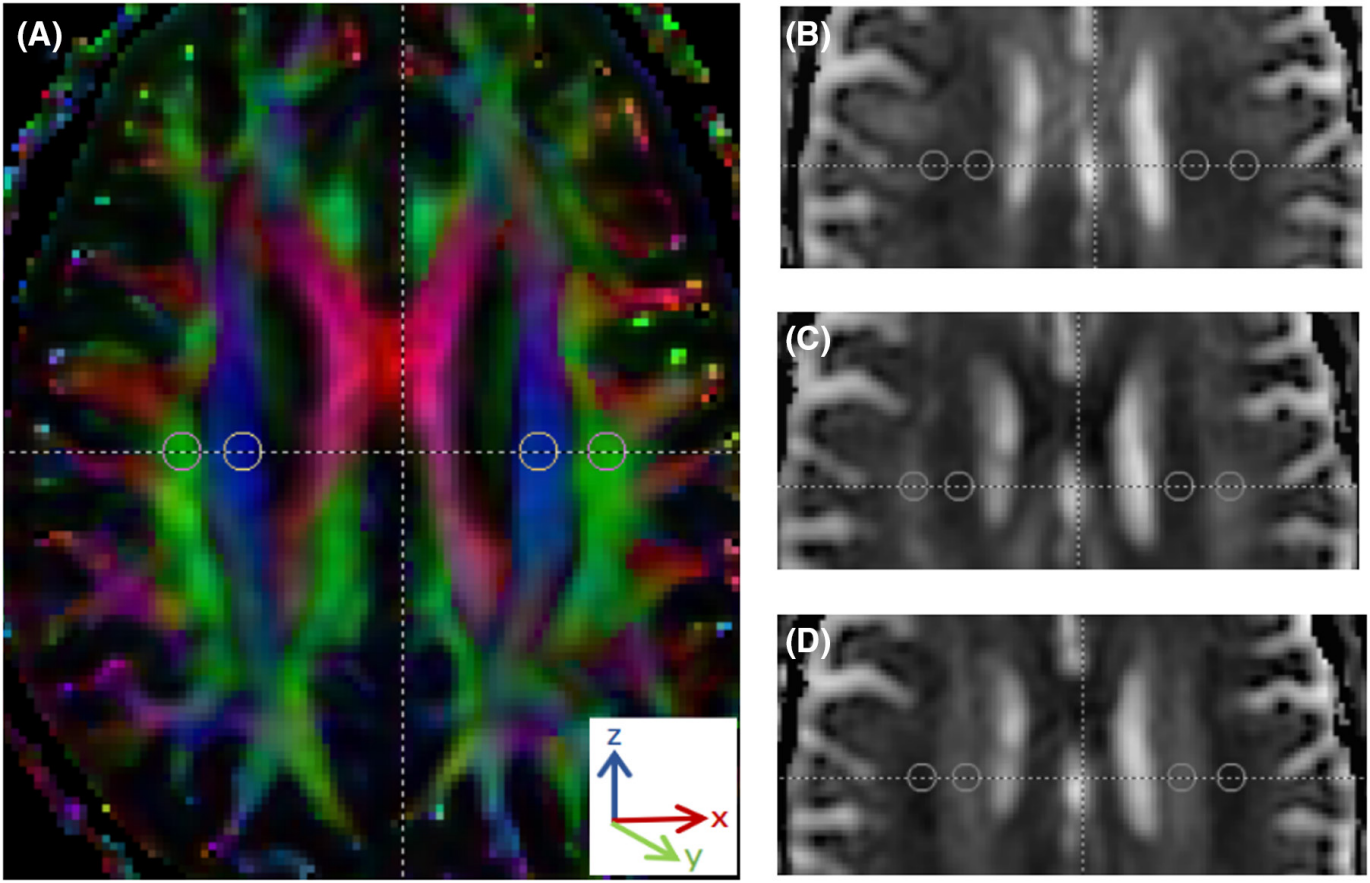
Calculation of index for diffusion tensor image analysis along the perivascular space (ALPS index). (Figure 1A). shows color‐coded fractional map exhibiting the direction of the projection fibers (blue; z‐axis), association fibers (green; y‐axis), and the subcortical fibers (red; x‐axis). Four 5‐mm‐diameter regions of interest (ROIs) were placed on bilateral projection fibers (blue area) and association fibers (green area) along the body of lateral ventricle, respectively. We recorded the diffusivities in the directions of the x‐axis (Dx, Figure 1B), y‐axis (Dy, Figure 1C), and z‐axis (Dz, Figure 1D) of ROIs on projection fibers and association fibers as Dxproj, Dyproj, Dzproj, Dxassoc, Dyassoc, Dzassoc, respectively. Then we calculated the unilateral ALPS index as [(Dxproj + Dxassoc) / (Dyproj + Dzassoc)] and obtained the average value of bilateral ALPS index

Glymphatic magnetic resonance imaging (MRI) is the classic method for evaluating glymphatic function directly. However, its application is limited due to the invasive intrathecal injection of gadolinium and repeated MRI scans with fixed time intervals.^21^ A non‐invasive method, the index for diffusion tensor image analysis along the perivascular space (DTI‐ALPS), has been proposed as a metric with possible sensitivity to glymphatic function.^22^ DTI‐ALPS method has been modified by Zhang et al. in 2021. They simplified the calculation method on only DTI, generated an ALPS index by this modified method (Figure 1), and verified the correlation between this ALPS index and the classical glymphatic function on glymphatic MRI.^23^ In recent years, the DTI‐ALPS method has been widely used in studies of cerebral small vascular disease (CSVD),^24^ Alzheimer's disease,^22^, ^25^ idiopathic hydrocephalus,^26^ diabetes mellitus,^27^ and aging.^25^, ^28^ Most studies focused on the relationship between ALPS index and cognition. ALPS index has also been revealed to be correlated with CSVD imaging markers, especially white matter hyperintensities (WMHs). Three studies used the DTI‐ALPS method to explore glymphatic activity in PD patients.^29^, ^30^, ^31^ McKnight et al.^31^ reported reduced ALPS index in PD patients compared to that in patients with essential tremor. Chen et al.^29^ and Ma et al.^30^ found that PD patients exhibited a reduced ALPS index than the healthy controls (HCs). However, the results of the correlations between the ALPS index and its related factors in different PD cohorts were controversial. Moreover, how the ALPS index interacts with sleep disorders and how it is involved in the disease severity of PD of different ages have not been reported yet.

The coupling of glymphatic function, sleep, age, and clearance of misfolded protein has prompted speculation that the pathophysiology of PD may relate to compromised glymphatic circulation. Therefore, the current study aimed to use the modified DTI‐ALPS method to explore the glymphatic activity and its related factors, including the severity of motor and non‐motor symptoms in PD patients. Age has been reported to be associated with different PD phenotypes by previous studies,^6^, ^7^ underpins which we hypothesized that glymphatic function plays different roles in the pathogenesis and progression of PD among patients of different age groups. So, associations between ALPS index and disease severity in PD patients of different age groups were also explored. Besides, we speculated there may be a link between risk factors of cardiovascular disease (CVD) and glymphatic dysfunction in PD for reasons as follows: CVD and its risk factors are common in the aged population^32^; arterial pulsation drives the glymphatic circulation^11^; and although CVD and PD share concordant risk factors including hypertension and diabetes mellitus, the relationship between PD and CVD and its risk factors are still undetermined.^32^ Hence, CVD risk factors and WMHs were also examined in this study.

## 2 MATERIALS AND METHODS

### 2.1 Participants

We retrospectively reviewed and analyzed the medical records and neuroimaging data of patients with idiopathic PD, recruited from the Department of Neurology of Guangdong Provincial People's Hospital from January 2018 to March 2021. PD diagnosis was made by 2 neurologists based on the United Kingdom PD Society Brain Bank Clinical Diagnostic Criteria. All participants were right‐handed Chinese natives, with comprehensive medical records, complete neuropsychological evaluations, and qualified DTI imaging data. Excluding criteria included parkinsonism symptoms induced by cerebrovascular disease, medications, trauma, encephalitis and poisoning, other neurodegenerative diseases, patients with dementia, and DTI data with obvious susceptibility‐induced distortions and Gibbs ringing artifacts. Finally, a total of 93 patients with idiopathic PD and 42 age‐ and sex‐matched HCs were included. The data concerning the age at disease of PD are highly variable in the literature. According to the World Health Organization (WHO), the age of 65 is recommended as the cutoff value for the elderly. Hence, we divided the patients with idiopathic PD with the age of 65 and above into the group of PD with older age, and the rest into PD with younger age, with 40 patients and 53 in each group. This study was approved by the Medical Ethics Committee of Guangdong Provincial People's Hospital [no. GDREC2018338H(R1)]. All participants were fully informed and provided written informed consent.

### 2.2 Clinical and neuropsychological assessments

All patients were off anti‐Parkinsonian medications for at least 12 hours prior to clinical assessments. Motor functions were assessed by the Movement Disorder Society‐sponsored revision of the Unified Parkinson's Disease Rating Scale part III (MDS‐UPDRS‐III) and Hoehn and Yahr (H&Y) stages. Neuropsychological evaluations were completed by one neuropsychologist blind to clinical information and MRI results, including the Hamilton anxiety scale (HAMA) and Hamilton depression scale‐24 items (HAMD‐24) for assessing psychological status; Parkinson's disease sleep scale 2nd version (PDSS‐2) for measuring sleep disorders^33^; Mini‐Mental State Examination (MMSE) for global cognition; picture alignment test from Wechsler Adult Intelligence Scale‐Chinese Revised (WAIS‐R) and the animal fluency test for executive functions; digit span and digit symbol tests from WAIS‐R for attention and working memory; intelligent memory and recognition tests from the Wechsler Memory Scale (WMS) for memory; vocabulary tests and similarities tests from WAIS‐R for language; block design tests from WIAS‐R and Rouleau clock drawing for visuospatial function. Patients with PD were divided into PD with normal cognition (PD‐NC) and PD with mild cognitive impairment (PD‐MCI) groups according to the Level II criteria of the Movement Disorder Society (MDS) Task Force guidelines,^34^ that is, impairment, represented by performance below 1.5 standard deviations (SD) norms, on at least two neuropsychological tests measuring the five cognitive domains. Besides, comprehensive information on demographics, personal history, and medical history was also collected.

### 2.3 Magnetic resonance imaging acquisition

All MRI examinations were performed after withdrawing from anti‐Parkinsonian medications for at least 12 h by using GE 3.0 T MR imaging system (Signa Excite HD GE Healthcare) with an 8‐channel head coil. During scanning, the subject was positioned supine in the framework of the scanner with foam padding to diminish motion artifacts. Sequences were as follows: T1‐weighted imaging (T1WI): repetition time/echo time (TR/TE) = 580/18 ms, T2‐weighted imaging (T2WI): TR/TE = 5100/130 ms, fluid‐attenuated inversion recovery (FLAIR): TR/TE = 9600/110 ms; matrix 320 × 192, FOV = 24 cm × 24 cm, slice thickness 5 mm, layer spacing 1 mm and a total of 25 layers. Diffusion tensor imaging (DTI): spinning‐echo echo‐planar imaging (SE‐EPI) sequence with TR/TE = 8000/76 ms, flip angle = 90; FOV = 256 × 256 mm^2^; slice thickness = 2.5 mm; voxel size = 2 × 2 × 3 mm^3^; and NEX =1. Images of 25 different nonlinear diffusion‐weighted gradient directions (b = 1000 s/mm^2^) and non‐diffusion‐weighted gradient direction (b = 0) were collected, each gradient was scanned for 60 layers, and a total of 1560 files were obtained. All data were saved in DICOM format.

### 2.4 Hypothesis and MRI analysis

At superior levels of the ventricular body, the deep medullary veins run perpendicularly to the ventricle wall.^35^ Thus, the direction of the perivascular space (PVS) is mostly in the right–left direction (x‐axis) on the axial plane, which is also vertical to the direction of the projection fibers (mostly in the z‐axis) as well as the association fibers (mostly in the y‐axis).^23^ At moderate‐to‐high b‐values typically used in DTI (i.e., b = 1000 s/mm^2^), diffusivity from flowing venous blood is suppressed. Therefore, the diffusivity along the x‐axis at regions with projection/association fibers will at least partly represent the diffusivity along the PVS, contributed by the glymphatic fluid transport. In the DTI‐ALPS model, Taoka et al.^22^ generated the ALPS index, quantified as the amount of diffusion along the x‐axis over the diffusion along the y‐axis and z‐axis, on DTI and susceptibility‐weighted imaging (SWI). Of note, the direction of medullary veins is homologous in most people, especially at the superior layer of the lateral ventricle body,^35^ making it possible to measure the diffusivity without the visualization of veins on SWI (Figure 1).

All DTI data were preprocessed as follows. The raw imaging data saved as DICOM format were first converted to NIFTI format by using the dcm2nii.exe toolkit in the MRIcroN software (https://www.nitrc.org/projects/mricron). At the same time, a b‐vector file and a b‐value file were created, which would be used to process and calculate the DTI data later. Then the converted data were denoised, head motion‐corrected, and eddy current‐corrected by using Automatic Image Registration toolkits on the DTI Studio (https://www.mristudio.org) and by following the instructions of the operation manual of DTI Studio. The preprocessed DTI data were further processed and calculated on DTI Studio. Details have been described in a recent study.^23^ Diffusivity maps in the direction of the x‐axis (right–left), y‐axis (anterior–posterior), z‐axis (inferior–superior), and color‐coded fractional anisotropy (FA) maps were created. At the location where the direction of the deep medullary veins was vertical to the lateral ventricle body, four 5‐mm‐diameter ROIs were placed on bilateral projection fibers and association fibers on FA map (Figure 1). Diffusivity in the directions of the x‐axis (Dx), y‐axis (Dy), and z‐axis (Dz) of ROIs on projection fibers and association fibers were recorded as Dxproj, Dyproj, Dzproj, Dxassoc, Dyassoc, Dzassoc, respectively. Then, ALPS index was calculated as [(Dxproj + Dxassoc)/(Dyproj + Dzassoc)] for each side. The average value of the bilateral ALPS index would be calculated as a measure of the glymphatic function of the basal ganglion area. A high ALPS index represents a good diffusivity along PVS, indicating a good glymphatic function. We tried to co‐register the diffusivity maps into a common FA map template and placed ROIs on the registered diffusivity maps, as proposed by Zhang et al^.^ ^23^ However, the majority of the ROIs covered the CSF area of the ventricle or the subarachnoid space on the diffusivity maps, whose diffusivities in all 3 directions were greatly impacted by those of free water. Hence, registration into a common template was abandoned. One trained neurologist blind to clinical data independently placed ROIs on each FA map and calculated ALPS index for each patient and re‐placed ROIs and re‐calculated ALPS index after 3 days interval for intra‐observer reliability analysis.

A trained neurologist blind to the clinical information reviewed T2WI and FLAIR images. Grading of deep white matter hyperintensities (DWMH) and periventricular white matter hyperintensities (PVWMH) was each assessed on a 0–3 point scale according to Fazekas, et al.^36^; DWMH was graded as 0 = absence, 1 = punctate foci, 2 = beginning confluence of foci, 3 = large confluent areas; PVWMH was graded as 0 = absence, 1 = cap, 2 = smooth halo, 3 = irregular and extending into the subcortical white matter. White matter hyperintensities (WMH) were a combination of DWMH and PVWMH.

### 2.5 Statistical analysis

Data were analyzed with SPSS Statistics version 23.0 (IBM cooperation). Internal consistency with Cronbach's α was conducted between intra‐observer analyses of ALPS index. The Kolmogorov–Smirnov test was performed to assess normality for the distribution of the continuous variables. Two‐sample *t*‐test, Mann–Whitney test, and chi‐square test were conducted to examine the clinical differences of normally distributed continuous variables, non‐normally distributed continuous variables, and categorical variables, between HC and PD groups, and between PD patients aged below 65 years old and PD patients aged 65 years old and above, respectively. In addition, with the ALPS index as the dependent variables, Pearson's correlation, Mann–Whitney test, and Spearman's correlation were performed to determine its correlations with the normally distributed continuous variables, dichotomous variables, non‐normally distributed continuous variables, and discrete variables, respectively, within all subjects, the PD group and the PD subgroups divided by the age of 65. And then, a stepwise multivariate linear regression analysis was carried out between the ALPS index and covariates with a *p‐*value below 0.1. A *p‐*value less than 0.05 is considered statistically significant.

## 3 RESULTS

### 3.1 Demographics and clinical data

We reviewed the medical records of patients with iPD from January 2018 to March 2021 and included 109 patients with neuroimaging sequences of good quality. Two patients were excluded for severe arteriostenosis of middle cerebral arteries, and 14 patients were excluded for incomplete records of clinical evaluations. Finally, a total of 93 participants with iPD and 42 HCs were included. The intra‐observer Cronbach's α of ALPS index was 0.980. Table 1 revealed details of the demographic and clinical data of the HC group, the PD group, and 2 age subgroups of PD patients divided by the age of 65 years old.

**TABLE 1:** Demographics and clinical data

	HC (*n* = 42)	PD (*n* = 93)	*p* value	PD with younger age (*n* = 53)	PD with older age (*n* = 40)	*p* value
Demographic characteristics						
Female sex, *n* (%)	23 (54.8%)	44 (47.3%)	0.461	26 (49.1%)	18 (45.0%)	0.834
Age, y, mean (SD)	61.52 ± 7.54	61.87 ± 8.52	0.668	55.94 ± 5.74	69.73 ± 4.02	<0.001^d^
Age onset, y, mean (SD)	NA	58.49 ± 8.82	NA	52.85 ± 6.73	65.98 ± 4.75	<0.001^d^
Disease duration, m, mean (SD)	NA	38.68 ± 34.88	NA	43.46 ± 39.71	32.34 ± 26.36	0.501
Medical history						
Hypertension, *n* (%)	11 (26.2%)	34 (36.6%)	0.324	13 (24.5%)	21 (52.5%)	0.009^e^
Diabetes mellitus, *n* (%)	2 (4.8%)	14 (15.1%)	0.148	7 (13.2%)	7 (17.5)	0.567
Dyslipidemia, *n* (%)	13 (31%)	24 (25.8%)	0.539	15 (28.3%)	9 (22.5%)	0.634
Hyperhomocysteinemia, *n* (%)	3 (7.1%)	7 (7.5%)	1.00	2 (3.8%)	5 (12.5%)	0.135
Personal history						
Smoking, *n* (%)	10 (23.8%)	15 (16.1%)	0.340	10 (18.9%)	5 (12.5%)	0.571
Drinking, *n* (%)	8 (19.0%)	13 (14.0%)	0.452	10 (18.9%)	3 (7.5%)	0.141
Education, y, mean (SD)	11.30 ± 3.32	9.34 ± 4.21	0.009^a^	9.04 ± 4.21	9.74 ± 4.22	0.374
Neuroimaging parameters						
Dx, e^‐4, mean (SD)	11.18 ± 1.19	10.58 ± 1.42	0.018^b^	10.70 ± 1.23	10.41 ± 1.63	0.338
Dy+Dz, e^‐4, mean (SD)	8.67 ± 1.10	9.44 ± 1.22	0.001^b^	9.10 ± 1.06	9.90 ± 1.28	0.001^f^
ALPS index, mean (SD)	1.31 ± 0.18	1.16 ± 0.18	<0.001^b^	1.22 ± 0.16	1.09 ± 0.18	<0.001^f^
PVWMH, M (IQR)	1 (0, 1)	1 (1, 1)	0.017^c^	1 (0, 1)	1 (1,2)	0.004^g^
DWMH, M (IQR)	0.5 (0, 1)	0 (0, 1)	0.924	0 (0, 1)	1 (0, 1)	0.018^g^
Motor functions and neuropsychological assessments						
MDS‐UPDRS‐III, mean (SD)	NA	35.04 ± 12.52	NA	35.23 ± 12.81	34.80 ± 12.30	0.741
PDSS‐2, M (IQR)	NA	12 (8, 19)	NA	12 (6.5, 16.5)	14 (10.2, 20.8)	0.021^g^
HAMA, M (IQR)	NA	9 (5, 14.5)	NA	8 (5, 12.5)	13 (7, 15)	0.083
HAMD‐24, M (IQR)	NA	11 (6, 18)	NA	9 (5, 13.5)	12 (7.2, 21.5)	0.033^g^
Hoeh & Yahr, M (IQR)	NA	2 (2,2.5)	NA	2 (2, 2.5)	2 (2, 2.5)	0.948
MMSE, M (IQR)	NA	28 (25, 29)	NA	28 (24, 29)	28 (25, 29)	0.987
MCI, *n* (%)	NA	44 (47.3%)	NA	22 (41.5%)	22 (55%)	0.215

*Note*: Significant difference defined as *p* < 0.05.

Abbreviations: ALPS, analysis along the perivascular space; DWMH, deep white matter hyperintensities; Dx, mean diffusivity along x‐axis in bilateral regions of projection fibers and association fibers; Dyproj+Dzassoc, mean diffusivity along y‐axis in bilateral regions of projection fibers and diffusivity along z‐axis in bilateral regions of association fibers; HAMA, Hamilton anxiety scale; HAMD‐24, Hamilton depression scale‐24 items; HCs, health controls; MCI, mild cognitive impairment; MDS‐UPDRS‐III, the Movement Disorder Sociaty (MDS) sponsored revision of the Unified Parkinson's Disease Rating Scale, part III (motor examination); MMSE, Mini‐Mental State Examination; MMSE, mini‐mental state examination; PD, Parkinson's disease; PDSS‐2, Parkinson's disease sleep scale 2nd version; PVWMH, periventricular white matter hyperintensities.

^a^ Mann–Whitney test between HCs and PD.

^b^ Two‐sample *t*‐test between HCs and PD.

^c^ Spearman's correlation between HCs and PD.

^d^ Mann–Whitney test between PD group below the age of 65 and PD group aged 65 and above.

^e^ Chi‐square test between PD group below the age of 65 and PD group aged 65 and above.

^f^ Two‐sample t‐test between PD group below the age of 65 and PD group aged 65 and above.

^g^ Spearman's correlation between PD group below the age of 65 and PD group aged 65 and above.

Participants in PD group presented with average age of 61.87 years (SD = 8.52), with a female/male ratio of 44/49. Among the PD patients, 34 (36.6%), 14 (15.1%), 24 (25.8%), 7 (7.5%), 15 (16.1%) and 8 (19%) had a history of hypertension, diabetes mellitus, dyslipidemia, hyperhomocysteinemia, smoking, and drinking, respectively. Twenty‐one (22.5%), 53 (56.9%), 15 (16.1%), and 4 (4.3%) scored 0, 1, 2 and 3 in the assessments of PVWMH, while 47 (50.5%), 39 (41.9%), 6 (6.4%) and 1 (1.1%) of the PD participants scored 0, 1, 2 and 3 in the assessments of DWMH, respectively. No significant differences were found in age, sex ratio, medical history, and scores of DWMH between the PD and HC groups. However, patients with PD presented with significantly lower ALPS index, lower education years, and higher PVWMH Fazekas scores than the HCs (1.16 ± 0.18 vs 1.31 ± 0.18, *t* = 4.41, df = 133, *p* < 0.001; 9.34 ± 4.21 vs 11.30 ± 3.32, *t* = 2.66, df = 133, *p* = 0.009; Spearman's rho = 0.21, *p* = 0.017; respectively). (Table 1).

The average onset age was 58.49 years (SD = 8.82) and the average disease duration was 38.68 months (SD = 34.88) among all the PD participants. The scores of MDS‐UPDRS part III were normally distributed, with an average of 35.04 (SD = 12.52). Scores of other scales were non‐normally distributed and were presented as median (inter‐quartile range, IQR) in Table 1. Of all the PD participants, 44 (47.3%) met the Level II criteria of PD‐MCI. No significant difference was found in the length of disease duration between the older PD group and the younger PD group, whereas the onset age of the older PD group was significantly older than that of the younger ones (65.98 ± 4.75 vs 52.85 ± 6.73, *z* = −8.235, *p <* 0.001). Compared with the younger PD group, the older PD group presented with more history of hypertension (χ^2^ = 7.69, *p* = 0.009), higher PVWMH Fazekas score (spearman's rho = 0.295, *p* = 0.004), higher DWMH Fazekas score (spearman's rho = 0.246, *p* = 0.018), and lower ALPS index (1.09 ± 0.18 vs 1.22 ± 0.16, t = 3.62, df = 91, *p* < 0.001). In addition, the older PD patients scored higher than the younger ones in scales of PDSS‐2, HAMA and HAMD‐24 (z = −2.316, *p* = 0.021; z = −1.731, *p* = 0.083; z = −2.130, *p* = 0.033; respectively). There were no significant differences in sex distribution, scores of MDS‐UPDRS part III, MMSE scores, the status of MCI, H&Y stage, personal history, and history of other medical conditions between the two age subgroups. (Table 1).

### 3.2 Correlation between ALPS index and demographics, disease status and WMH

**FIGURE 2:**
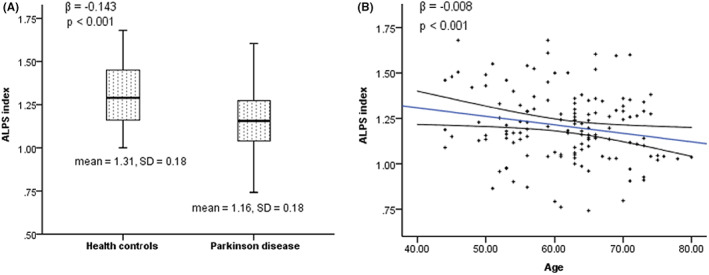
(A) Correlation between ALPS index and disease condition of PD. (B) Correlation between ALPS index and age among all subjects. The β value and p value were obtained from stepwise multivariate regression analysis. Trend lines depict linear regression with 95% confidence intervals

Table 2 presented factors related to ALPS index revealed by univariate analysis in different population groups. PD participants demonstrated a significantly lower ALPS index than the HCs as mentioned before, which remained significant after being adjusted by age, sex, medical history, and WMH Fazekas score in multivariate linear regression analysis (*β* = −0.143, *p* < 0.001, Figure 2A). Among all the participants, ALPS index was inversely associated with age (Spearman's rho = −0.338, *p* < 0.001) and the association remained significant in multivariate linear regression analysis (*β* = −0.008, *p* < 0.001, Figure 2B). Univariate analysis revealed negative correlations between ALPS index and history of diabetes mellitus (*z* = −2.158, *p* = 0.031), PVWMH Fazekas score (Spearman's rho = −0.269, *p* = 0.002), DWMH Fazekas score (spearman's rho = −0.195, *p* = 0.024) and WMH Fazekas score (Spearman's rho = −0.195, *p* = 0.002), which were found insignificant after adjustment of age, sex and the status of PD by multivariate linear regression analysis.

**TABLE 2:** Univariate analysis with the ALPS index as dependent variable

	All subjects (*n* = 135)		PD subjects (*n* = 93)		PD with younger age (*n* = 53)		PD with older age (*n* = 40)	
	*r/z*	*p* value	*r/z*	*p* value	*r/z*	*p* value	*r/z*	*p* value
Age	−0.338	<0.001	−0.386	<0.001	−0.197	0.158	−0.231	0.152
Female sex	−1.754	0.079	−0.031	0.975	−0.391	0.695	−0.816	0.415
PD	−3.943	<0.001	NA	NA	NA	NA	NA	NA
Age onset	NA	NA	−0.339	0.001	−0.214	0.123	0.014	0.932
Disease duration	NA	NA	−0.167	0.110	−0.002	0.988	−0.325	0.041
History of hypertension	−1.741	0.082	−1.891	0.059	−0.682	0.495	−0.636	0.524
History of diabetes mellitus	−2.158	0.031	−1.708	0.088	−1.287	0.198	−0.908	0.364
History of dyslipidemia	−0.675	0.501	−0.299	0.765	−0.948	0.343	−1.247	0.212
History of hyperhomocysteinemia	−0.811	0.417	−1.587	0.112	−0.140	0.899	−0.716	0.474
History of smoking	−0.133	0.894	−0.480	0.631	−0.796	0.426	−0.348	0.728
History of drinking	−0.076	0.940	−0.033	0.973	−0.568	0.570	−1.720	0.085
PVWMH	−0.269	0.002	−0.204	0.050	−0.178	0.203	0.036	0.825
DWMH	−0.195	0.024	−0.228	0.028	−0.220	0.114	−0.052	0.750
WMH	−0.260	0.002	−0.246	0.018	−0.231	0.096	−0.011	0.945
MDS‐UPDRS‐III	NA	NA	−0.109	0.297	0.035	0.805	−0.315	0.048
PDSS‐2	NA	NA	−0.260	0.012	0.223	0.108	−0.183	0.250
HAMA	NA	NA	−0.105	0.315	−0.031	0.823	−0.033	0.840
HAMD‐24	NA	NA	0.060	0.958	0.108	0.441	0.105	0.445
Hoeh & Yahr	NA	NA	−0.155	0.139	−0.132	0.346	−0.192	0.234
MMSE	NA	NA	−0.166	0.111	−0.240	0.083	−0.120	0.459
PD‐MCI	NA	NA	−0.390	0.973	−0.776	0.438	−0.734	0.463

*Note*: Sex, PD, medical history, personal history, and PD‐MCI were compared by Mann–Whitney test. MDS‐UPDRS‐III scores were analyzed by Pearson's correlation. Age, age onset, disease duration, PVWMH, DWMH, WMH, and scores of other scales were analyzed by Spearman's correlation.

Significant difference defined as *p* < 0.05.

Abbreviations: ALPS, analysis along the perivascular space; DWMH, deep white matter hyperintensities; Dx, mean diffusivity along x‐axis in bilateral regions of projection fibers and association fibers; HAMA, Hamilton anxiety scale; HAMD‐24, Hamilton depression scale‐24 items; MDS‐UPDRS‐III, the Movement Disorder Society (MDS) sponsored revision of the Unified Parkinson's Disease Rating Scale, part III (motor examination); MMSE, Mini‐Mental State Examination; MMSE, mini‐mental state examination; PD, Parkinson's disease; PD‐MCI, Parkinson’s disease with mild cognitive impairment; PDSS‐2, Parkinson's disease sleep scale 2nd version; PVWMH, periventricular white matter hyperintensities; WMH, white matter hyperintensities.

**FIGURE 3:**
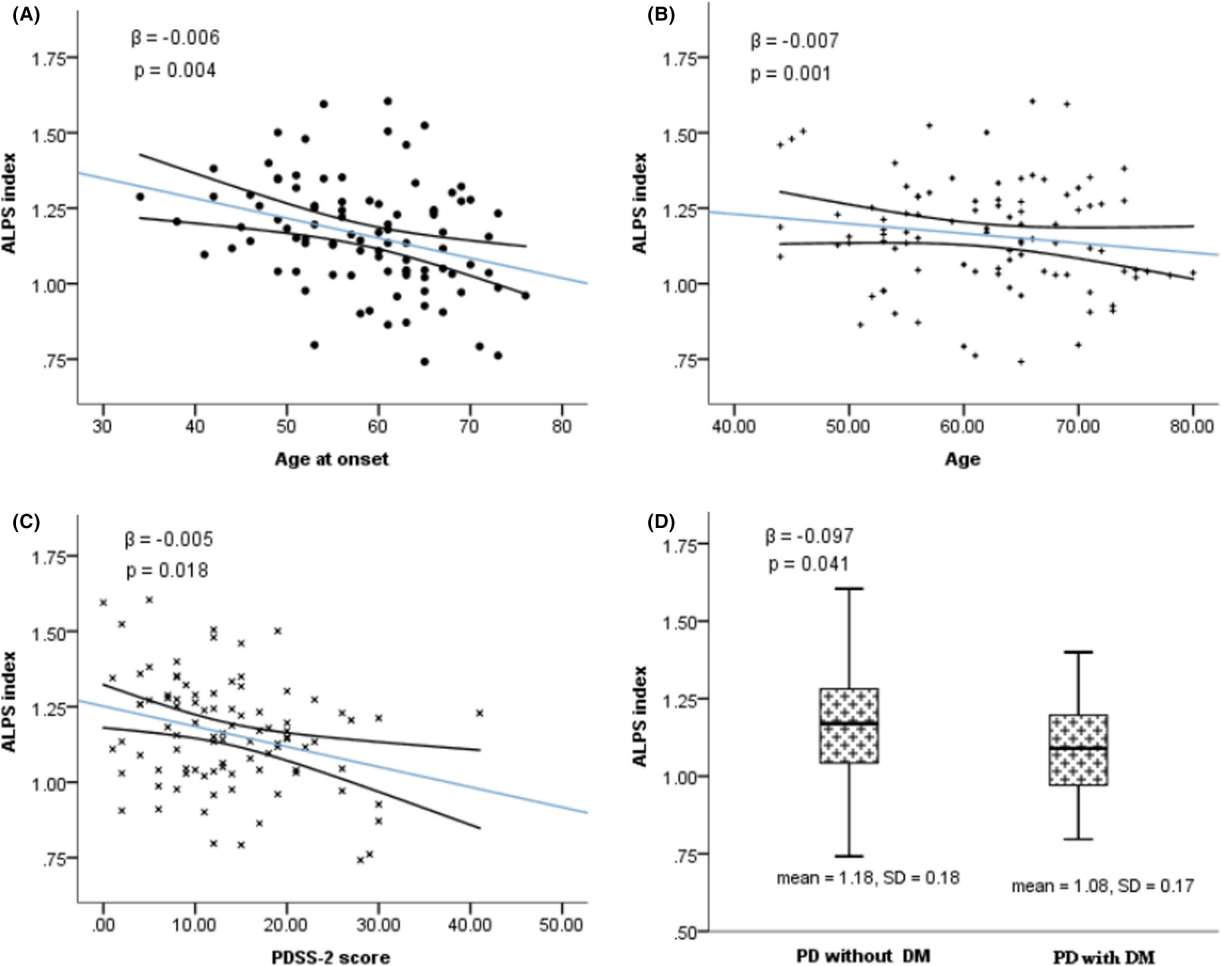
(A) Correlation between ALPS index and onset age of PD. (B) Correlation between ALPS index and age in PD group. (C) Correlation between ALPS index and PDSS‐2 scores in PD group. (D) Correlation between ALPS index and history of diabetes mellitus in PD group. Trend lines depict linear regression with 95% confidence intervals. The β value and p value of (A) were obtained from stepwise multivariate regression analysis model with onset age, history of diabetes mellitus, PDSS‐2 scores, history of hypertension and white matter hyperintensities Fazekas score as covariates. The β value and p value of (B), (C), (D) were obtained from stepwise multivariate regression analysis model with age, history of diabetes mellitus, PDSS‐2 scores, history of hypertension and WMH Fazekas score as covariates. ALPS, analysis along the perivascular space; PDSS‐2, Parkinson's disease sleep scale 2nd version; PD, Parkinson's disease; DM, diabetes mellitus

**FIGURE 4:**
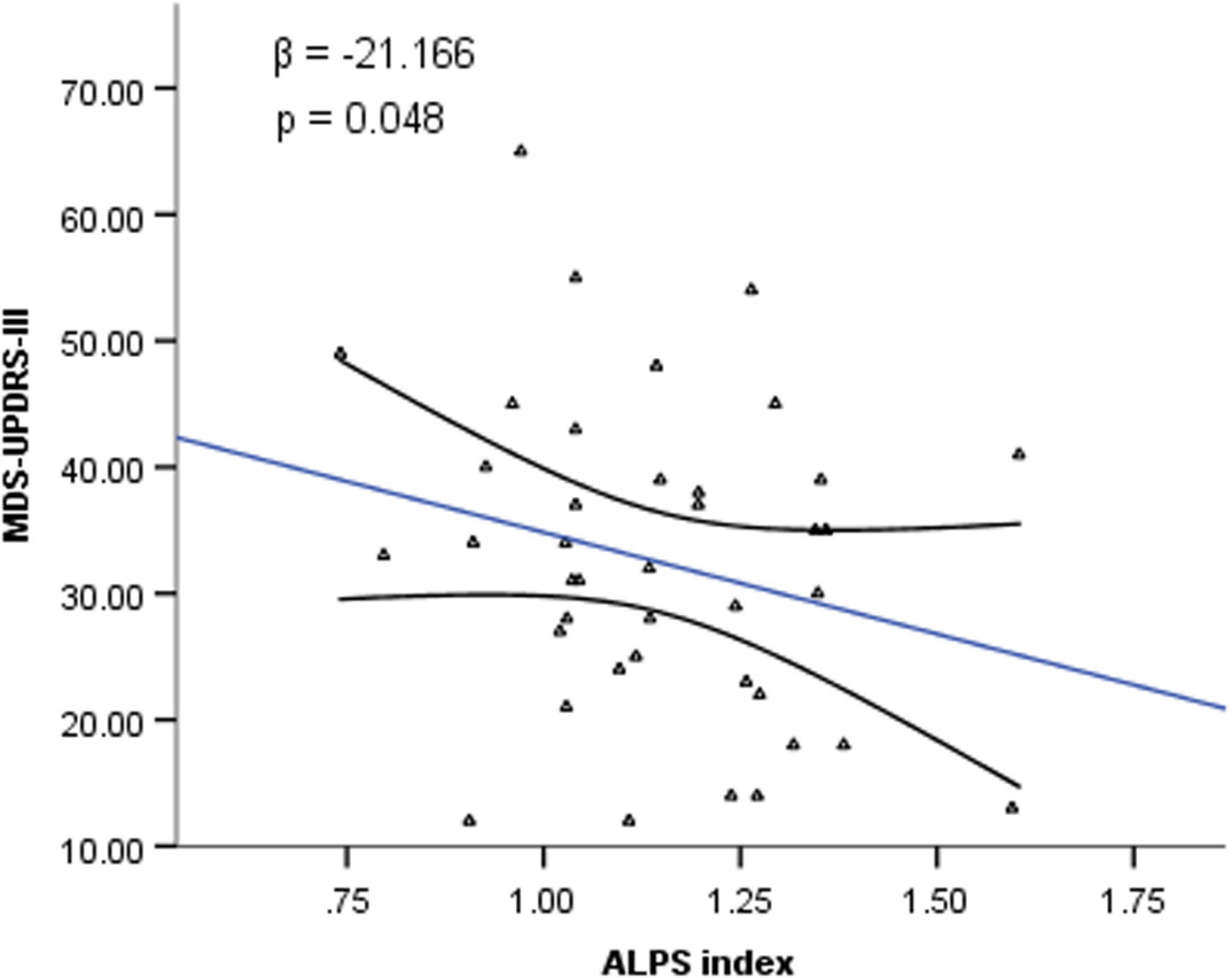
Correlation between MDS‐UPDRS‐III scores and ALPS index in PD group aged 65 and above. Trend lines depict linear regression with 95% confidence intervals. The β value and p value were obtained from stepwise multivariate regression analysis. ALPS, analysis along the perivascular space; MDS‐UPDRS‐III, the Movement Disorder Society (MDS) sponsored revision of the Unified Parkinson's Disease Rating Scale, part III (motor examination)

In the PD group, negative correlations between ALPS index and DWMH Fazekas score (spearman's rho = −0.228, *p* = 0.028) and WMH Fazekas score (spearman's rho = −0.246, *p* = 0.018) were observed but were of no significance adjusted by age, history of hypertension, and history of diabetes mellitus. Stepwise multivariate linear regression analysis revealed negative relationships between ALPS index and age of onset (*β* = −0.006, *p* = 0.004, Figure 3A), age (*β* = −0.007, *p* = 0.001, Figure 3B), PDSS‐2 scores (*β* = −0.005, *p* = 0.018, Figure 3C) and history of diabetes mellitus (*β* = −0.097, *p* = 0.041, Figure 3D). These relationships still had trends but were of no statistical significance in the PD patients below the age of 65. Subgroup analysis among the PD patients aged 65 and above revealed negative relationships between ALPS index and disease duration (Spearman's rho = −0.325, *p* = 0.041), and between ALPS index and scores of MDS‐UPDRS part III (Spearman's rho = −0.315, *p* = 0.048). Multivariate linear regression analysis with scores of MDS‐UPDRS part III as the dependent variable and with disease duration as a covariate revealed a negative relationship between MDS‐UPDRS part III scores and ALPS index in the PD group aged 65 and above (*β* = −21.166, *p* = 0.048, Figure 4).

## 4 DISCUSSION

The current study exhibited reduced ALPS index in PD patients compared with the HCs and revealed inverse correlations between the ALPS index and clinical information including age at disease onset, age at MRI scanning, sleep disorders indicated by high PDSS‐2 scores, and history of diabetes mellitus in the PD group. In addition, with disease duration as the covariate, subgroup analysis revealed a negative correlation between ALPS index and the severity of motor symptoms measured by MDS‐UPDRS part III in the older PD group, rather than in the younger one. Using the non‐invasive DTI‐ALPS method to measure glymphatic activity, this study contributed to accumulating evidence of the involvement of glymphatic dysfunction in Parkinson's disease, especially in those with advanced age and indicated that compromised glymphatic function may be the reasonable mechanism that interlinks aging and PD. With the measure of comorbidities, including CVD risk factors and WMHs, and multivariate linear regression test with the comorbidities and demographic data as covariates, results related to age and age subgroups should be interpreted as relatively directly related to ALPS index.

The identification of glymphatic system has promoted an understanding of how the brain maintains its homeostasis by clearing and draining proteins and toxic metabolites from brain parenchyma through the directionally polarized perivascular pathway.^12^, ^37^ Reduced ALPS index measures reflect less robust fluid transport in the direction of the perivascular pathway and compromised glymphatic clearance. Although much is unknown about how abnormal proteins accumulate, aggregate, and propagate in proteinopathies, stagnant fluid flow in the perivascular spaces, decreased glymphatic clearance and elevated concentration of extracellular abnormal proteins are believed to be greatly involved in this process.^38^ Albeit majorities of relevant evidence came from studies focused on beta‐amyloid (Aβ) protein with AD models, study with PD animal models verified that injection of α‐synuclein preformed fibrils into bilateral striatum contributed to impaired lymphatic drainage,^39^ which in turn exacerbated motor and cognition deficits.^13^ In addition, impaired CSF flow and drainage dysfunction have been reported in PD patients in various studies using different imaging methods.^29^, ^30^, ^31^, ^39^ Data of the current study support the hypothesis that compromised glymphatic circulation is an underlying mechanism of α‐synuclein accumulation, contributing to the pathogenesis and progression of PD.

PD is typically considered age‐related. Previous studies demonstrated that an up to 85% reduction of CSF inflow of large tracers in aged wild‐type mice^15^ and that contrast clearance in human brain tissue was inversely correlated to age in all subjects with intrathecal injection of gadolinium.^16^ In the current PD group, a correlation was identified between age and decreased glymphatic circulation, indicating that compromised glymphatic function and the resulted decreased clearance of metabolites and toxic proteins underlie the mechanism from the natural aging process to PD. Age and disease duration are the two important timescales of disease progression.^5^ However, the age at disease onset of different PD patients can vary by decades. Previous studies demonstrated that advanced age at onset was associated with a severe phenotype of PD with accelerated disease progression,^6^, ^7^, ^8^ consistent with decreased CSF α‐synuclein^7^ and increased CSF lactic acid^8^ in PD patients with advanced age at onset. These results suggest that PD patients of different ages at onset, probably with different pathophysiological mechanisms involved, should be treated differently. The current study contributed to this hypothesis by revealing an association between reduced ALPS index and older age at onset. We took it further by examining the ALPS index and its related factors in different age subgroups. No significant difference in disease duration, but a significant one in age at disease onset was observed between the two age subgroups. The severity of motor symptoms as well as disease duration was associated with ALPS index in the older PD group rather than in the younger one. Moreover, reduced ALPS index contributed to more severe motor symptoms in the older patients independent of the disease duration. It is reasonable to infer that glymphatic dysfunction is greatly involved in the process of disease progression of PD patients with older age at onset, while an alternative pathophysiological mechanism less affected by glymphatic function is suggested in the younger ones.

Both PD patients and the aged share a high prevalence of sleep disorders. The importance of sleep in ensuring brain homeostasis has gained wide attention. Xie et al.^13^ confirmed that cortical interstitial space dilates during sleep and thus promotes convective flow and clearance of Aβ in the brains of adult mice. Fultz et al.^14^ revealed that CSF flow was coupled with slow‐wave neural activity and verified robust activity of glymphatic clearance in non‐rapid eye movement sleep in human brains. The PDSS‐2 scale is a validated clinical rating scale that assesses the frequency of sleep disturbances over the past week, with higher PDSS‐2 scores indicating more sleep disorders in PD patients.^33^ Our data revealed an independent negative association between ALPS index and PDSS‐2 scores and support the conclusion that sleep disorders contribute to impaired glymphatic function in PD patients, suggesting glymphatic dysfunction as a possible underlying mechanism that interlinks aging, sleep disorders and PD. Comorbidities, that is, hypertension, diabetes mellitus, and white matter hyperintensities (WMHs) are correlated with age, respectively. Previous studies reported reduced glymphatic activity in patients with these diseases.^23^, ^27^ Low‐grade inflammation and overproduction of reactive oxygen species may interlink the state of these diseases with PD, but their relationship remains controversial.^32^ The current study provides supportive evidence that diabetes mellitus contribute to the impaired glymphatic function. However, the possible negative associations between ALPS index and hypertension and between ALPS index and WMH were insignificant after being adjusted by age. In addition, we did not reveal any association between ALPS index and MCI state in PD patients or between ALPS index and MMSE scores in PD patients of the current study, consistent with a previous study.^31^

The results of the current study should be interpreted in view of several limitations. Firstly, the ALPS index was an indirect measure of glymphatic activity in the basal ganglion area. Albeit the correlation between the ALPS index and classical glymphatic function measured by Glymphatic MRI has been verified by a recent study,^23^ results related to this noninvasive method should be interpreted with caution. This study provides evidence for the feasibility of the noninvasive DTI‐ALPS method in exploring glymphatic function in PD patients. However, whether this ALPS index can be used as a measure of glymphatic activity of the global brain needs further research. Secondly, with a restricted sample size in this retrospective study, we failed to provide a reasonable cutoff value for age‐at‐onset groups to explore the pathophysiological processes related to glymphatic function. A cohort study exploring the relationship between temporal changes of ALPS index and age at disease onset will provide stronger evidence for understanding underlying pathophysiology. Thirdly, although the current study suggested an association between sleep disturbances and impaired glymphatic activity in patients with PD, to make a profound understanding of how glymphatic function is involved in the pathogenesis of sleep disturbances and PD, prospective cohort work focusing on glymphatic activity and α‐synuclein aggregation in sleep disturbances that precede clinical diagnosis of PD is necessary. Lastly, defects related to the processing of DTI data must be pointed out. The current study did not apply the SWI to locate the medullary veins, as proposed by the classic DTI‐ALPS method,^22^ to delineate the regions of projection fibers and association fibers accurately. Moreover, head circumferences were not corrected and may account for the gender differences in ALPS index among all participants in this study. Future studies should take these factors into account when using this DTI‐ALPS method.

## 5 CONCLUSION

This study demonstrated reduced ALPS index, a potential noninvasive measure of compromised glymphatic function, in PD patients and provided evidence supporting the association between glymphatic dysfunction and aging, sleep disorders, and diabetes mellitus. In addition, the different findings related to age subgroups denote glymphatic activity may be differently involved in the pathophysiology of PD patients of different ages. These results support the conclusion that compromised glymphatic transport may be the mechanism underlying the pathogenesis and progression of PD, especially in those aged 65 and above. Special attention should be paid to glymphatic function in the aged ones, especially those with sleep disorders and diabetes mellitus.

## AUTHOR CONTRIBUTIONS

Xin Cai and Yuhu Zhang contributed to conception and organization of the research project; Piao Zhang and Limin Wang contributed to collection of clinical data of participants; Xin Cai, Chentao He, and Zhenzhen Chen contributed to MRI image collection, processing, and data analysis; Kun Nie and Yihui Qiu contributed to statistical analysis; Ping Jing, Lijuan Wang, Zhenzhen Chen, and Yuhu Zhang contributed to review and critique of the statistical analysis; Xin Cai contributed to writing of the first draft; Ping Jing, Lijuan Wang, and Yuhu Zhang contributed to the final approval for submission.

## FUNDING INFORMATION

This work was supported by the National Natural Science Foundation of China (No.82071419); High‐level Hospital Construction Project (No.DFJH201907); Supporting Research Funds for Outstanding Young Medical Talents in Guangdong Province (No.KJ012019442); Medical Scientific Research Foundation of Guangdong Province, China (No. A2019141).

## CONFLICT OF INTEREST

All authors declare no actual or potential conflict of interest.

## Data Availability

Origin data of the current study are available from the corresponding author on reasonable request.
